# A Simple and Rapid Turn On ESIPT Fluorescent Probe for Colorimetric and Ratiometric Detection of Biothiols in Living Cells

**DOI:** 10.1038/s41598-017-03901-8

**Published:** 2017-06-29

**Authors:** Yi Wang, Meiqing Zhu, Erkang Jiang, Rimao Hua, Risong Na, Qing X. Li

**Affiliations:** 1 Department of Science of Pesticides, School of Resources and Environment, Anhui Agricultural University, No. 130 Changjiang West Road, Hefei, 230036 China; 2 Collaborative Innovation Center of Henan Grain Crops, National Key Laboratory of Wheat and Maize Crop Science, College of Plant Protection, Henan Agricultural University, Wenhua Road No. 95, Zhengzhou, 450002 China; 3 Department of Molecular Biosciences and Bioengineering, University of Hawaii at Manoa, 1955 East-West Road, Honolulu, HI 96822 USA; 4 State Key Laboratory of Tea Plant Biology and Utilization, School of Life Sciences, Anhui Agriculture University, No. 130 Changjiang West Road, Hefei, 230036 China

## Abstract

Biothiols, such as cysteine (Cys), homocysteine (Hcy), and glutathione (GSH), play a key role in an extensive range of physiological processes and biological functions. Therefore, the selective and sensitive detection of intracellular thiols is important for revealing cellular function. In this study, ethyl 2-(4-(acryloyloxy)-3-formylphenyl)-4-methylthiazole-5-carboxylate (**NL-AC**) was designed and synthesized as a colorimetric and ratiometric fluorescent probe that can be utilized to rapidly, sensitively and selectively detect biothiols in physiological media. The fluorescence intensity of this probe using the three target biothiols at a concentration of 20 equiv. of the probe increased by approximately 6~10-fold in comparison to that without the biothiols in aqueous solution. The limits of detection (LOD) for Cys, Hcy and GSH were 0.156, 0.185, and 1.838 μM, respectively. In addition, both ^1^H-NMR and MS analyses suggested the mechanism of fluorescence sensing to be excited-state intramolecular proton transfer (ESIPT). The novel colorimetric and ratiometric probe is structurally simple and offers detection within 20 min. Furthermore, this probe can be successfully applied in bioimaging. The results indicate high application potential in analytical chemistry and diagnostics.

## Introduction

Important thiol-containing amino acids and biomolecules, including cysteine (Cys), homocysteine (Hcy) and glutathione (GSH), contain a mercapto group and play crucial roles in various physiological processes in living systems, such as maintaining biological thiol homeostasis, post-translational modifications, biocatalysis, metal binding and xenobiotic detoxification. Recently, these biothiols have received much research attention^1–4^. Changes in the levels of biothiols are related to various diseases. For example, there is a close association of Cys with neurotoxicity^5^, fat loss, skin lesions, slow growth in children, liver damage and muscle weakness^6–8^. Hcy deficiency leads to the risk of inflammatory bowel disease, Alzheimer’s disease^9^ and osteoporosis^10^. GSH has also been directly linked to diseases such as cancer, Parkinson’s disease and Alzheimer’s disease^11^. Due to their significant biological roles, the ability to detect and quantify such biothiols under physiological conditions is very important for academic research and disease diagnosis.

Many analytical techniques for the detection of the three biothiols have been employed, including high-performance liquid chromatography (HPLC), immunoassays, capillary electrophoresis (CE), electrochemical assays, UV-Vis spectroscopy, Fourier transform infrared spectroscopy (FT-IR), mass spectrometry (MS) and fluorescence spectroscopy^12^. Among these methods, fluorescence sensing is highly appropriate due to its advantage of high selectivity, high sensitivity, low detection limit, ease of use and great potential application in living cell imaging with fluorescent probes^7, 13–24^. In the development of different types of fluorescent sensors or probes, ratiometric fluorescent probes have attracted increasing attention based on the ratio of emission intensity from two well-resolved wavelengths, which can provide a built-in correction of background effects and increase the dynamic range of the fluorescence measurement.

Due to the advantages of salient properties such as the extremely large fluorescence Stokes shift and ultra-fast reaction rate, excited-stated intramolecular proton transfer (ESIPT) compounds have attracted attention for their potential applications in the field of optics^25^. To date, some ratiometric probes for the detection of biothiols undergoing an ESIPT process have been reported^12, 26–31^. 2-(2′-Hydroxyphenyl)benzothiazole (HBT) is an ESIPT dye; however, only a few ratiometric probes for selective and sensitive detection of biothiols on the basis of the structure of HBT or its analog have been studied^28, 30^. Therefore, a new strategy for the design and development of ratiometric fluorescent probes for the selective detection of biothiols is highly desirable.

**Figure 1:**
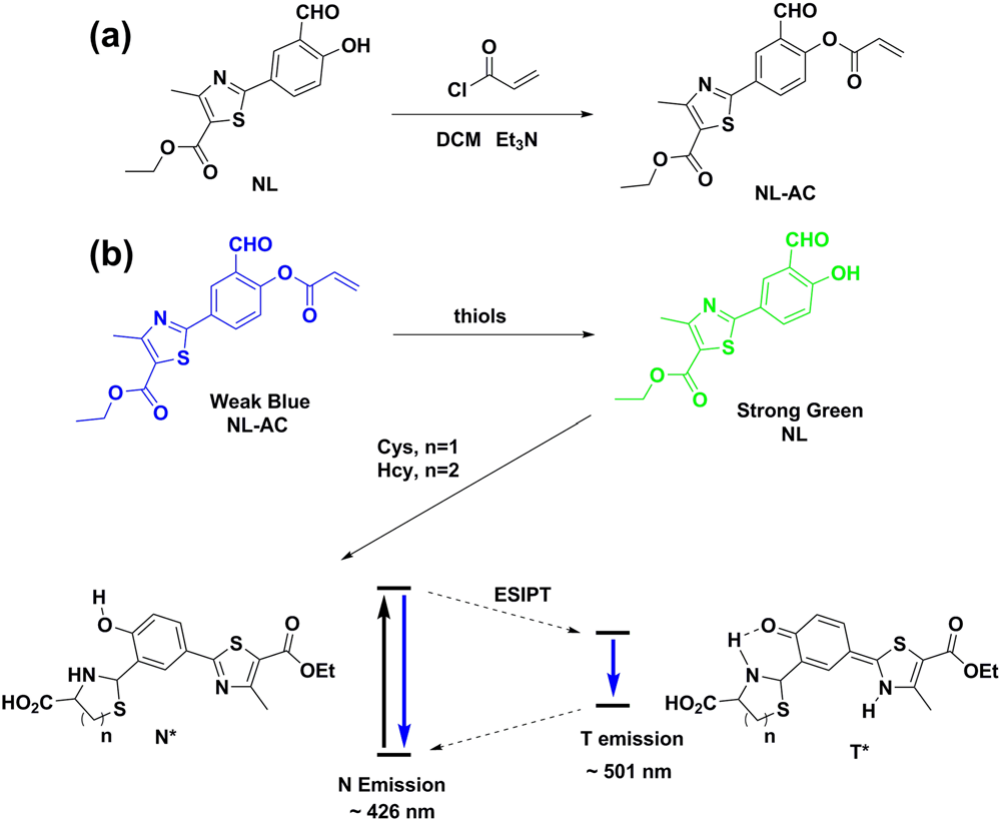
(**a**) Synthesis of the **NL-AC** probe and (**b**) chemical structures of **NL-AC** and biothiols along with a schematic representation of the ESIPT process of **NL** with Cys/Hcy.

In the present work, we combined the above strategies to design and synthesize the compound ethyl 2-(4-(acryloyloxy)-3-formylphenyl)-4-methylthiazole-5-carboxylate (**NL-AC**) with two reaction sites (Fig. 1), based on the fluorescent probe ethyl 2-(3-formyl-4-hydroxyphenyl)- 4-methylthiazole-5-carboxylate (**NL)** ^31^. As a novel colorimetric and ratiometric fluorescent biothiol probe, **NL** is structurally similar to HBT. The key features of the novel colorimetric and ratiometric probe **NL-AC** include high sensitivity, high selectivity, rapid detection (approximately 20 min) and suitability for live imaging. This probe undergoes an ESIPT process, as confirmed by ^1^H-NMR and MS spectra. This study using **NL-AC** may provide useful information for further research on the rational design of ESIPT biothiol probes.

## Results and Discussion

### Design and Synthesis of the Probe

**NL-AC** was conveniently synthesized via the acylation of **NL** with acryloyl chloride. Introduction of the acrylate group, which has a strong electron-withdrawing ability and is typically used as a functional trigger moiety to detect biological thiols^3, 32–37^, could result in a shift in fluorescence and cause ratiometric fluorescence changes. The cyclization and cleavage reaction with the targets changes the fluorescence as the electron-withdrawing groups depart. The structure of **NL-AC** was confirmed by ^1^H-NMR, ^13^C-NMR and HRMS.

### Optical Response of NL-AC to Biothiols

**Figure 2:**
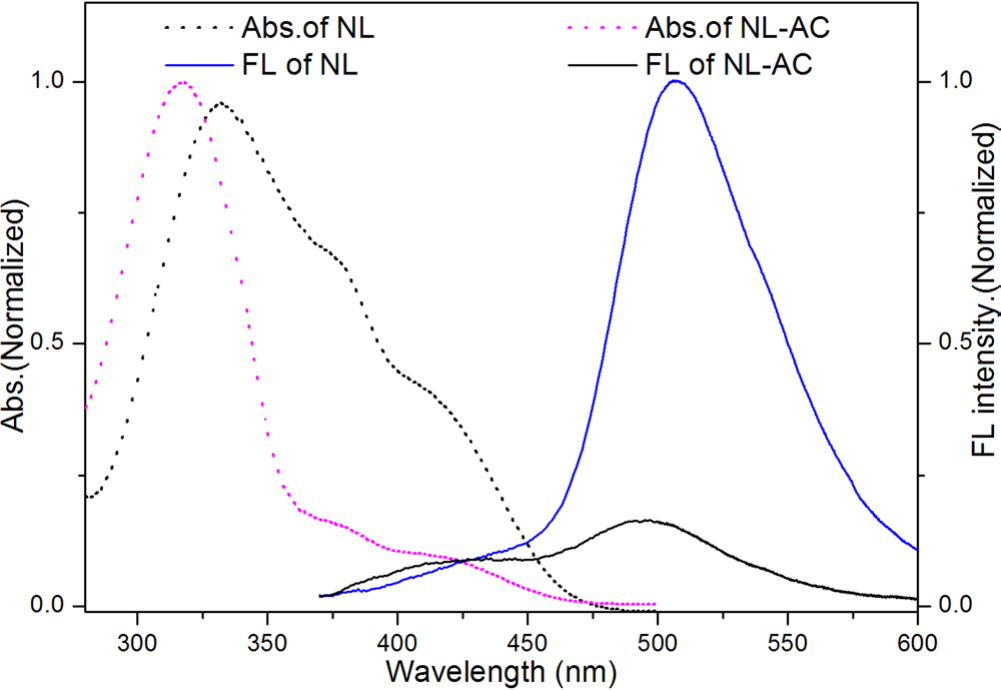
Absorption (dashed lines) and fluorescence (solid lines) spectra of **NL-AC** (10 µM) and **NL** (10 µM) in DMSO-H_2_O (8:2, *v*/*v*) solution (pH 7.4, 10 mM HEPES buffer).

As shown in Fig. 2, **NL-AC** exhibited an absorption peak at 316 nm, whereas **NL** showed two different absorption peaks at 327 and 380 nm. When excited at 336 nm, **NL-AC** exhibited only one weak emission peak at approximately 501 nm.

**Figure 3:**
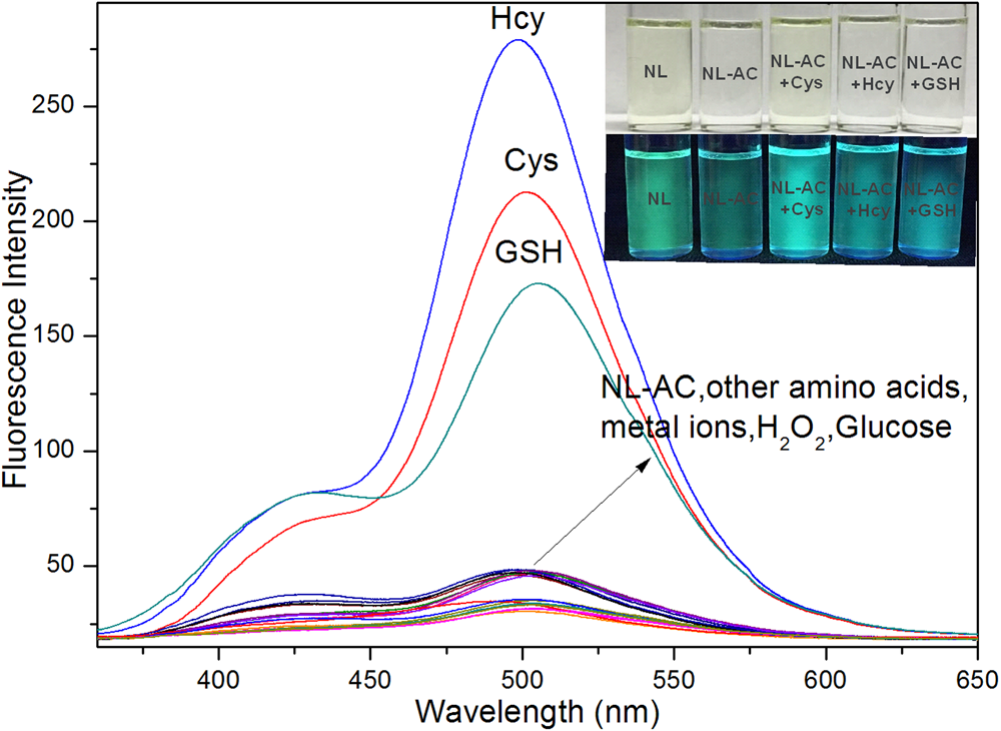
Fluorescence spectra of **NL-AC** (10 µM) with various analytes (200 µM) in HEPES buffer solution (DMSO/HEPES = 8:2, pH 7.4, λ_ex_ = 336 nm, slit: 5.0 nm/5.0 nm). The inset is a photograph of **NL-AC** without and with biothiols under ambient lighting (upper) or UV irradiation (lower).

We then investigated the optical sensing behavior of **NL-AC** toward the three biothiols, Cys, Hcy and GSH, individually using 10 µM of **NL-AC** in dimethyl sulfoxide (DMSO)-H_2_O (8:2, *v*/*v*) solution (pH 7.4, 10 mM HEPES buffer) at ambient temperature. The addition of biothiols to **NL-AC** produced two different emission bands at 426 and 501 nm, due to the normal isomer (N* emission) and tautomer (T* emission) of **NL**, respectively (Fig. 1). An intense blue-green emission from the “**NL-AC** + biothiols” sample was observed, which indicated that the hydroxyl group was released by addition of biothiols with **NL-AC**. Thus, the ESIPT process enabled a shift of the emission signal to a longer wavelength, and **NL** was generated. The ratio of two fluorescent bands can determine the analytes more accurately via minimization of the background signal instead of the absolute intensity of one band. Therefore, the new emission peak can be utilized for ratiometric fluorescent measurement. As shown in Fig. 3, the color of the solution in the bottle changed from colorless to yellow, which was clearly recognizable under ambient lighting. In addition, the fluorescence color also changed from weak blue to green by 365-nm UV irradiation. These results indicated that the probe was sensitive for the detection of the three biothiols.

### Mechanism of NL-AC for the Detection of Biothiols

**Figure 4:**
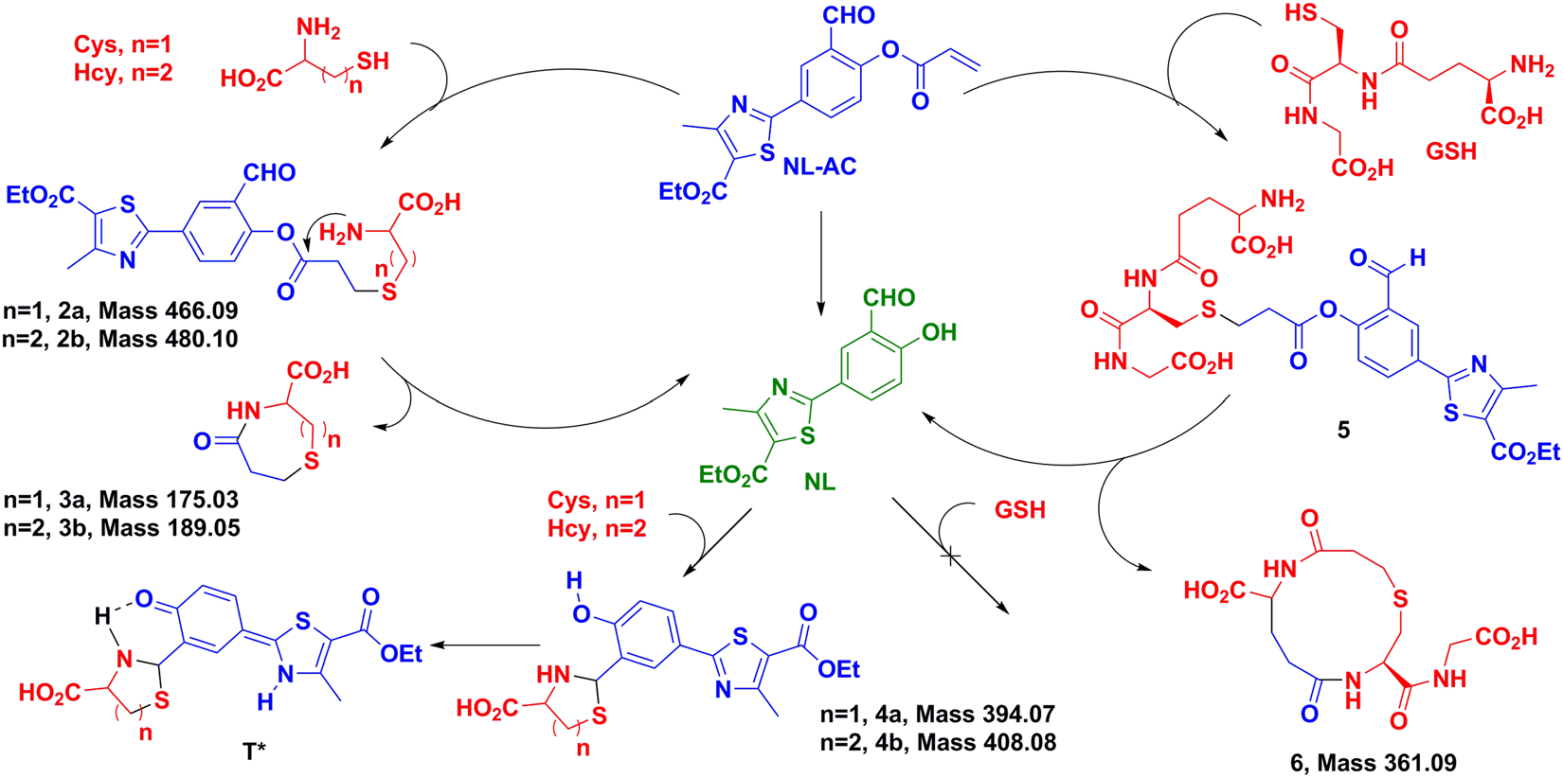
Proposed reaction mechanism of **NL-AC** with Cys, Hcy and GSH.

**Figure 5:**
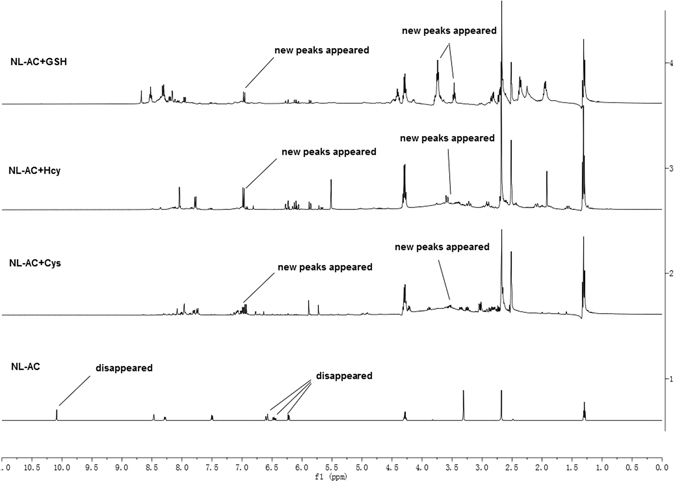
^1^H NMR spectrum of **NL-AC** in *d*_6_-DMSO and the resulting spectrum after the addition of Cys, Hcy or GSH.

A proposed reaction mechanism between **NL-AC** and biothiols based on the literature is depicted in Fig. 4 ^30, 32, 38^. The acrylate and aldehyde groups comprise two reactive sites. The series of cascade reactions may involve cyclization, rearrangement and condensation. First, the characteristic alkenyl proton disappeared completely between *δ* 6.2 and *δ* 6.7 ppm (Fig. 5), suggesting a reaction between the thiol and alkene, which generated thioethers **2a**, **2b** and **5**. As the reaction proceeds, it can additionally undergo an intramolecular cyclization to produce the desired compounds, **NL**, **3a**, **3b** and **6**. The presumptive products were confirmed by UPLC-MS/MS spectral analyses, based on the peaks at m/z 291.92, 175.97, 190.26 and 362.32 (calcd. [M + H]^+^ 292.06, 176.04, 190.05 and 362.10, Sup. Fig. 2). The product (**NL**) that was released from **NL-AC** still had a free aldehyde group. We have previously reported that **NL** can detect Cys and Hcy over GSH and other amino acids^31^. Similarly, in this system, the reaction products of **NL** with Cys and Hcy can also be verified. The aldehyde proton disappeared at approximately δ 10.09 ppm, and the thiazolidine methane proton appeared at approximately δ 5.8 ppm (Fig. 5). Furthermore, the [M + H]^+^ ions of products **4a** and **4**b were 394.96 and 408.89, respectively (calcd. [M + H]^+^ 395.07 and 409.09, Sup. Fig. 2).

### Selective Response of the NL-AC Probe to Biothiols

**Figure 6:**
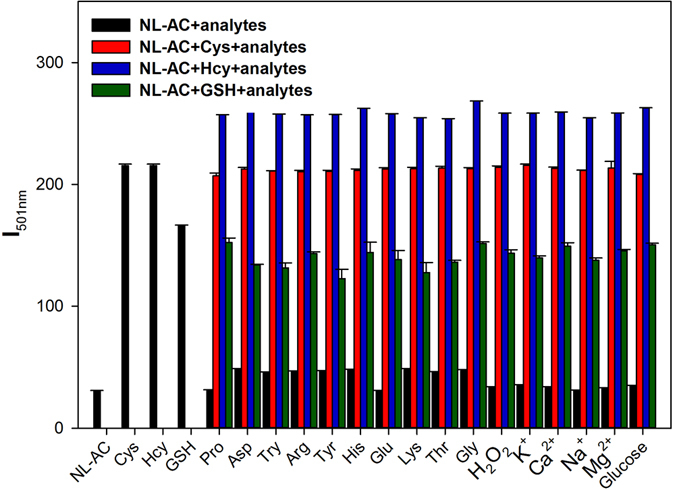
Fluorescence intensity (I_501nm_) of **NL-AC** (10 μM) without or with biothiols (200 μM) in the presence of various analytes (200 μM) in HEPES buffer solution (DMSO/HEPES = 8:2, pH 7.4, λ_ex_ = 336 nm, slit: 5.0 nm/5.0 nm).

The selectivity of **NL-AC** for biothiol measurements was recorded in DMSO/HEPES solution containing each of the 16 interference analytes, with the addition of the analytes of biothiols. These interference chemicals included Pro, Asp, Try, Arg, Tyr, His, Glu, Lys, Thr, Gly, H_2_O_2_, K^+^, Ca^2+^, Na^+^, Mg^2+^ and glucose, which each of them was at a concentration of 20-fold of the **NL-AC** probe. Only the biothiols triggered a markedly peak at 501 nm, whereas the other chemicals caused negligible effects on the fluorescence intensity of **NL-AC**. Competitive experiments were performed by treating **NL-AC** with biothiols in conjunction of other amino acids, metal ions and analytes, which showed negligible interference (Figs 3 and 6). Therefore, **NL-AC** demonstrated high selectivity toward biothiols in the presence of related amino acids, metal ions and other analytes.

### Reaction Time and Effect of pH

**Figure 7:**
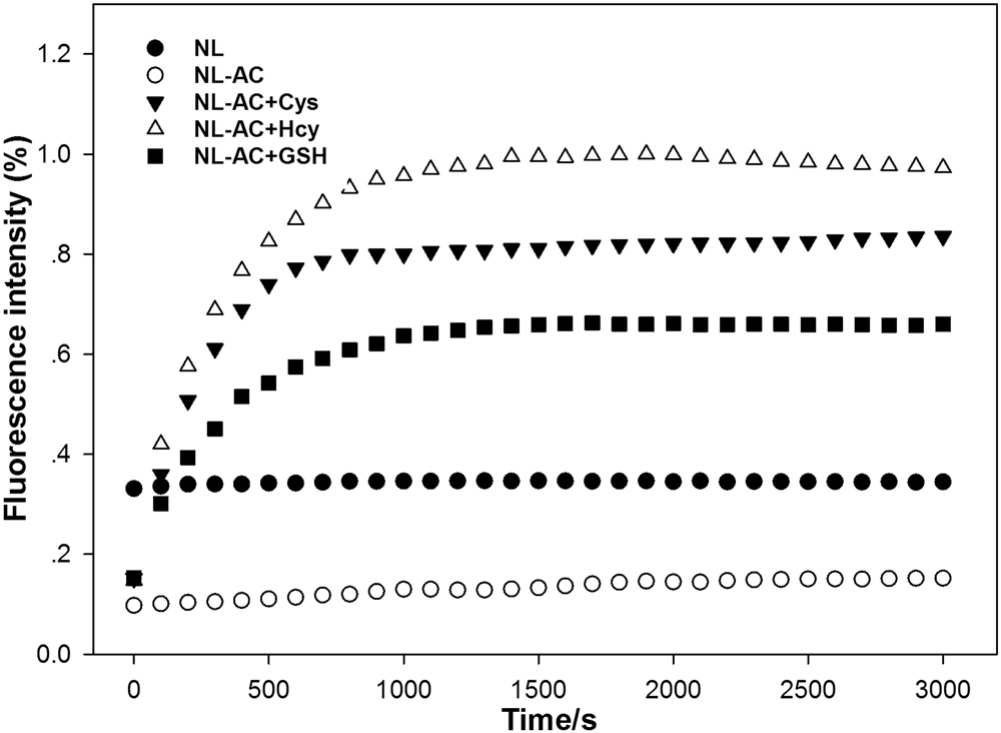
Time-dependent fluorescence intensity (%) at 501 nm of **NL-AC** (1 × 10^−5^ M) without and with Cys (2 × 10^−4^ M), Hcy (2 × 10^−4^ M) and GSH (2 × 10^−4^ M) in HEPES buffer solution (DMSO/HEPES = 8:2, pH 7.4, λ_ex_ = 336 nm, slit: 5.0 nm/5.0 nm).

To investigate the accurate reaction time of **NL-AC** with biothiols, the fluorescence intensity (%) at λ_em_ 501 nm was measured at varying periods of time after the addition of Cys, Hcy and GSH to the **NL-AC** solutions. As shown in Fig. 7, the reaction of **NL-AC** with Cys or Hcy was nearly completed within 20 min at ambient temperature. Compared to the probes reported (Sup. Table 1), **NL-AC** provides a rapid response to Cys with satisfactory sensitivity. Such an advantage makes **NL-AC** a suitable candidate to detect endogenous mitochondrial Cys level changes. Although some previously generated probes can detect biothiols at high concentrations, such as 30 equiv. or even more than 1000 equiv. of the probe^39–42^, **NL-AC** required only 1 equiv. to achieve accurate measurements of the biothiols in the present study.

We also investigated the effect of pH on the detection of **NL-AC** for biothiols. **NL-AC** could sensitively react with Cys in the range of pH 2-8, and the fluorescence changes were very apparent in this pH range (Sup. Fig. 4). In the absence of Cys, **NL-AC** was sufficiently stable in the pH range of 2.0 to 7.4. Weak alkaline conditions may promote the reaction of **NL-AC** with Cys. The ester groups of the probe might be hydrolyzed under strong alkaline conditions, causing a change in the structure of **NL-AC**. Consequently, the stable fluorescence response toward Cys at a pH value of approximately 7.4 was favorable for the detection experiments. **NL-AC** can function in the physiological pH range, which would be present in a cellular environment.

### Quantification of Biothiols

**Figure 8:**
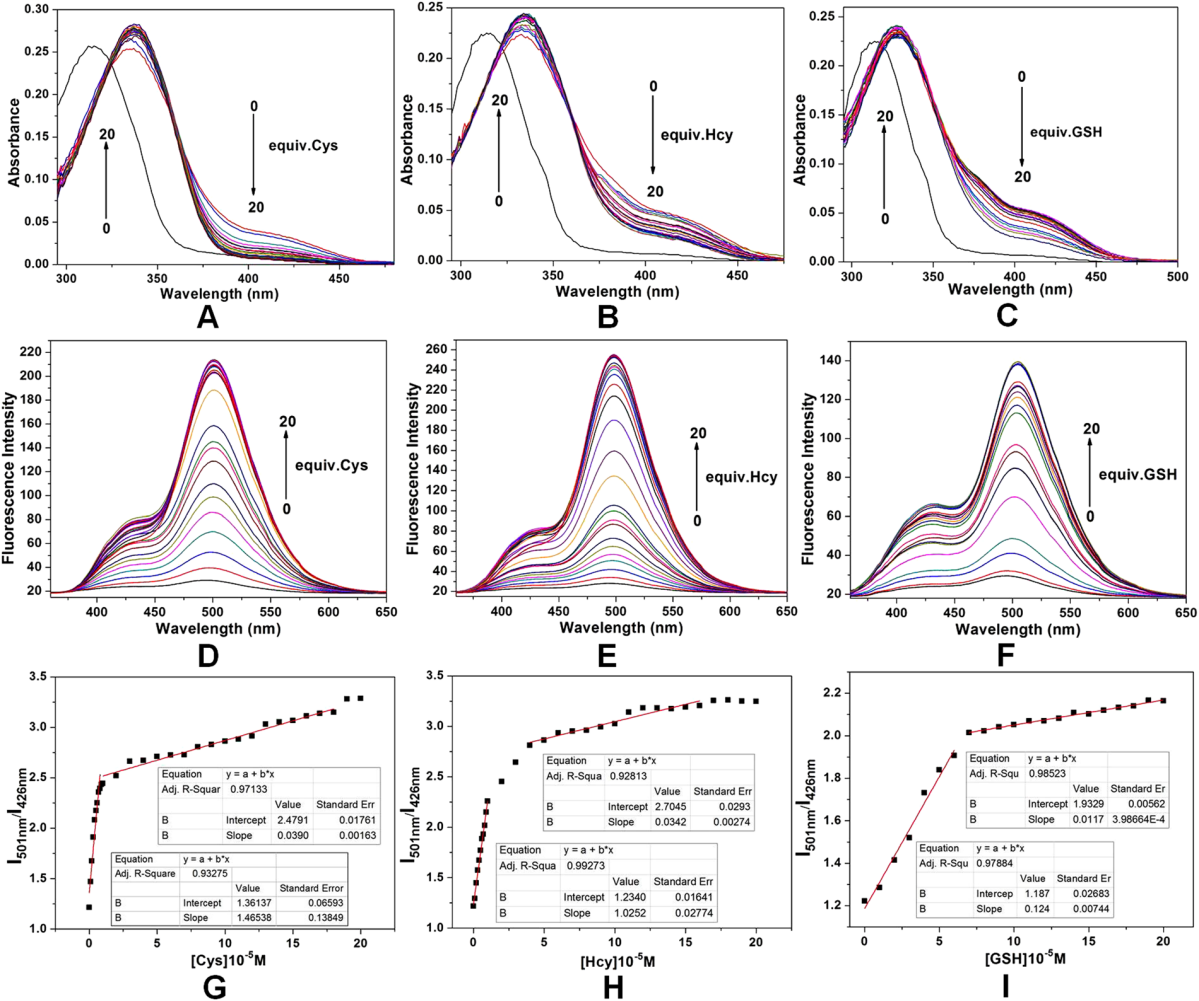
(**A**–**C**) UV-Vis absorption spectra of **NL-AC** (10 μM) with Cys/Hcy/GSH (0, 0.1, 0.2, 0.3, 0.4, 0.5, 0.6, 0.7, 0.8, 0.9, 1, 2, 3, 4, 5, 6, 7, 8, 9, 10, 11, 12, 13, 14, 15, 16, 17, 18, 19 and 20 equiv.) in HEPES buffer solution (DMSO/HEPES = 8:2, pH 7.4); (**D**–**F**) Fluorescence spectra of **NL-AC** (10 μM) with Cys/Hcy/GSH (0, 0.1, 0.2, 0.3, 0.4, 0.5, 0.6, 0.7, 0.8, 0.9, 1, 2, 3, 4, 5, 6, 7, 8, 9, 10, 11, 12, 13, 14, 15, 16, 17, 18, 19 and 20 equiv.) in HEPES buffer solution (DMSO/HEPES = 8:2, pH 7.4, λ_ex = _336 nm, slit: 5.0 nm/5.0 nm); (**G**–**I**) A plot of the ratiometric response (I_501_/I_426_) of **NL-AC** (10 μM) against Cys/Hcy/GSH equiv. is shown; the data represent means ± standard error (bars) (n = 3).

The addition of biothiols to the solution with the **NL-AC** probe led to increasing intensity of the absorption peak, which was red-shifted at 336 nm, as well as the simultaneous emergence of a new absorption peak at 380 nm (Fig. 8A–C). The fluorescence intensity of the probe solution increased at λ_em_ 501 nm with increasing content of biothiols. When the biothiols were at 20 equiv. of **NL-AC**, both the fluorescence intensity and ratio of the absorption intensities at 501 and 426 nm (I_501_/I_426_) were near their maximum values (Fig. 8D–F), indicating performance superior to that of other probes^39, 43^. In addition, a good linear correlation (R^2^ = 0.9328) existed between the fluorescence intensity and Cys concentration at a range of 0–1 µM. The limit of detection (LOD) of Cys was 0.156 µM (Fig. 8G) based on a S/N of 3. A similar response of the **NL-AC** probe to Hcy/GSH was also observed (Fig. 8H and I). In this case, the LOD values were 0.185 µM and 1.838 µM, respectively, based on the linear relationship (R^2^ = 0.9927, R^2^ = 0.9788) between the fluorescence intensity and Hcy/GSH concentration in the range of 0–1 µM. Compared to the reported probes^23, 44–46^, **NL-AC** demonstrated a much lower LOD for the qualitative analysis of Cys, Hcy and GSH (the intracellular level for Cys: 30–200 µM; Hcy: 5.0–13.9 µM; GSH: 1–10 mM^47^), which is useful for to the analysis of human plasma samples^48^.

### Effect of DMSO on the Detection of Cys

The effect of DMSO on the fluorescence intensity of the free **NL-AC** probe and the behavior of the probe toward Cys were also examined. The ratio of **NL-AC** rose with increasing content of DMSO (Sup. Fig. 3). The DMSO buffer solutions were optimized on the basis of the fluorescence signal-to-background ratio of **NL-AC** in the presence or absence of Cys. Therefore, the optimized solution consisted of a mixture of DMSO and HEPES buffer (8:2, *v*/*v*, pH 7.4) and was used in all subsequent experiments.

### Application of NL-AC

**Figure 9:**
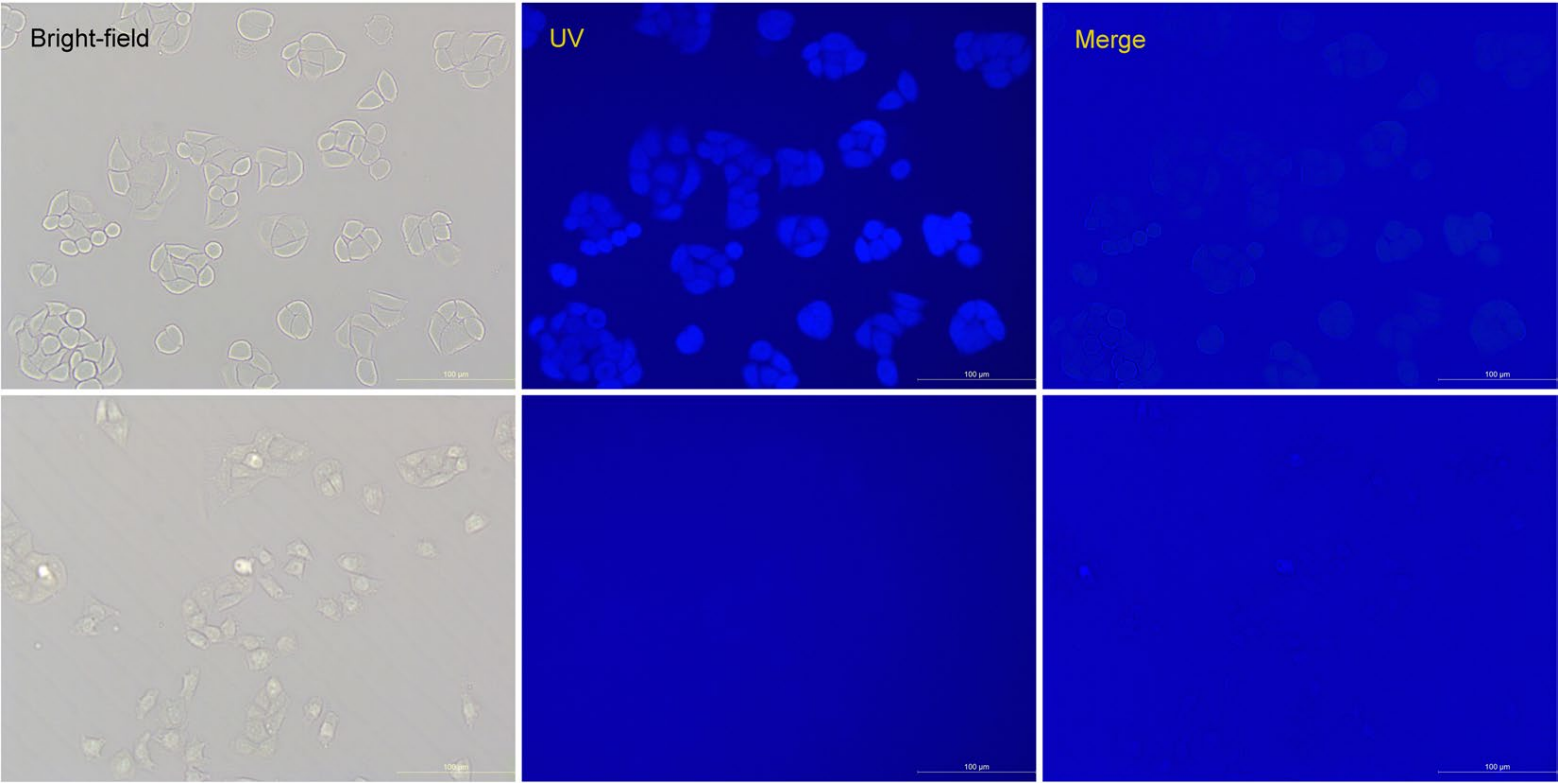
Fluorescence images of HeLa cells. Upper: HeLa cells were treated with 20 μM **NL-AC** for 0.5 h; Lower: HeLa cells were preincubated with 1 mM NEM for 2 h and then with 20 μM **NL-AC** for 0.5 h.

To further confirm the usability of **NL-AC** for the fluorescence imaging of biothiols, we performed experiments with living cells. HeLa cells were incubated with **NL-AC** (Fig. 9). The fluorescence intensity was correlated with the concentration of the probe (Sup. Fig. 5). To demonstrate the specificity of the probe to biothiols, HeLa cells were preincubated with a thiol-blocking agent (N-ethylmaleimide, NEM) to remove the endogenous biothiols in cells and were then incubated with the probe for 0.5 h. No fluorescence was observed (Fig. 9). The results revealed that the probe was specific for biothiols in living cells.

In summary, a novel colorimetric and ratiometric fluorescent probe, **NL-AC**, was designed, synthesized and demonstrated to distinguish biothiols from other amino acids at physiological pH values. The probe exhibited high sensitivity and selectivity toward biothiols both in aqueous solution and living cells. The fluorescence intensity of the probe was enhanced 6~10-fold upon the addition of biothiols at the concentration of 20 equiv. of the probe. **NL-AC** can sensitively detect Cys at concentrations ranging from 0.156 to 200 µM, Hcy at from 0.185 to 200 µM and GSH from 1.838 to 200 µM.

## Methods

### Materials

Commercial reagents were obtained from J&K (Beijing, China), Alfa Aesar (Ward Hill, MA, USA) and Sigma-Aldrich (St. Louis, MO, USA). Deionized water was purified with a Milli-Q Plus System from Millipore (Billerica, MA, USA) was used throughout all experiments. Nuclear magnetic resonance (NMR) spectra were recorded with a 600-MHz spectrometer (Agilent Technologies, Inc., Santa Clara, CA, USA). Mass spectra were recorded with a UPLC/XevoTQ MS/MS spectrometer (Waters, Milford, MA, USA) and an Agilent Accurate-Mass-Q-TOF MS 6520 system equipped with an Electrospray ionization (ESI) source. Reactions were stirred magnetically and monitored by thin-layer chromatography (TLC). Flash chromatography was performed using silica gel 60 (200–300 mesh). UV-Vis spectra were recorded with a UV-1800 spectrophotometer (Shimadzu, Kyoto, Japan), with quartz (1-cm path length). All fluorescence measurements were performed on a 970CRT fluorescence spectrophotometer (Inesa, Shanghai, China) equipped with a xenon lamp source. All pH values were measured with a PHS-25 digital pH meter (Shanghai REX Instrument Factory, Shanghai, China).

### Synthesis of NL-AC

A mixture of **NL** (1.5 g, 5.1 mM) and acryloyl chloride (1.398 g, 15.3 mM) was dissolved in 30 mL of anhydrous dichloromethane. After addition of triethylamine (1.5 mL) was added to the solution at 0 °C, the reaction mixture was warmed to ambient temperature overnight with stirring. The organic solvent was removed *in vacuo*, and the product in the residue was then purified over silica gel using hexane/ethyl acetate as the eluent (6:1, *v*/*v*) to obtain a white solid (1.045 g, 3.03 mM, 58.8% yield). ^1^H-NMR (600 MHz, DMSO-*d*_6_): *δ* 10.09 (s, 1 H), 8.47 (d, *J* = 2.5 Hz, 1 H), 8.28 (m, 1 H), 7.50 (d, *J* = 8.4 Hz, 1 H), 6.63–6.55 (m, 1 H), 6.46 (m, 1 H), 6.24–6.20 (m, 1 H), 4.28 (m, 2 H), 2.68 (s, 3 H), 1.29 (t, *J* = 7.1 Hz, 3 H). ^13^C-NMR (151 MHz, DMSO-*d*_6_): *δ* 189.81, 167.11, 164.06, 161.64, 160.78, 152.58, 134.92, 133.46, 130.88, 129.73, 128.84, 127.52, 125.42, 122.58, 61.77, 17.60, 14.54. HRMS: *m/z* calcd for C_17_H_15_NO_5_S [M + H]^+^ 346.0744; found, 346.0748.

### General UV-Vis and Fluorescence Spectra Measurements

Amino acids (Cys, Hcy, GSH, arginine (Arg), aspartic acid (Asp), glutamic acid (Glu), glycine (Gly), histidine (His), lysine (Lys), proline (Pro), threonine (Thr), tryptophan (Try), tyrosine (Tyr)), and cations (K^+^, Ca^2+^, Na^+^, Mg^2+^ and Zn^2+^) were all dissolved in deionized water at the concentration of 5 mM. Stock solutions of **NL-AC** (12.5 µM) were prepared in DMSO. The stock solutions of different analytes were diluted to a series of concentrations in HEPES buffer with deionized water. Test solutions were prepared by the addition of 2,400 µL of the **NL-AC** stock solution and an appropriate aliquot of test analyte into a 3-mL volumetric cuvette. Thus, the final concentration of **NL-AC** was 10 µM. The resulting solution was shaken well and incubated for 20 min at ambient temperature prior to use. Fluorescence and UV-Vis spectra were both obtained using DMSO and HEPES buffer (8:2, *v*/*v*) at pH 7.4.

### Cell Culture and Imaging

HeLa cells were cultured in Dulbeccos modified Eagle’s medium (DMEM). **NL-AC** was dissolved in DMSO at the storage concentration of 10 mM. Cells were cultured in 24-well culture plates for 12 h. HeLa cells were washed from the culture medium, incubated with 5, 10 and 20 µM (final concentration) of probe solution for 0.5 h at 37 °C, and then washed three times with phosphate-buffered saline (PBS). Cells were imaged under UV light with a Leica DMi8 inverted fluorescence microscope (Wetzlar, Germany).

## Electronic supplementary material

Supporting Information
