# Supplementary material for: A Simple and Rapid Turn On ESIPT Fluorescent Probe for Colorimetric and Ratiometric Detection of Biothiols in Living Cells
Source: Sci Rep. 2017 Jun 29;7:4377. doi: 10.1038/s41598-017-03901-8 (PMC5491497; doi:10.1038/s41598-017-03901-8)
Supplement: Supplementary file 1 — Supporting Information [file 41598_2017_3901_MOESM1_ESM.pdf]

Supplementary Information:

A Simple and Rapid Turn On ESIPT Fluorescent Probe for Colorimetric and Ratiometric Detection of Biothiols in Living Cells

Yi Wang, Meiqing Zhu, Erkang Jiang, Rimao Hua*, Risong Na* and Qing X. Li

Supplementary Figures

^{1}H-NMR of NL-AC

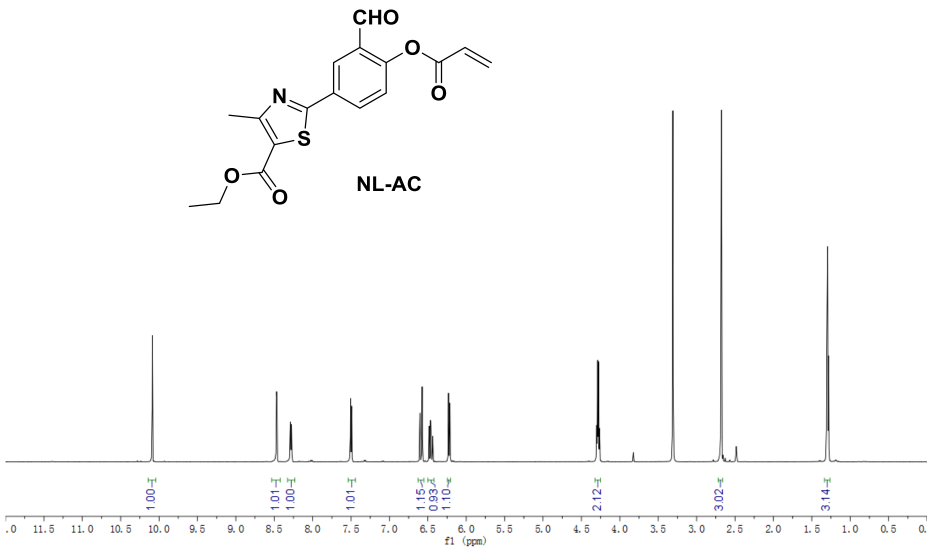

^{13}C-NMR of NL-AC

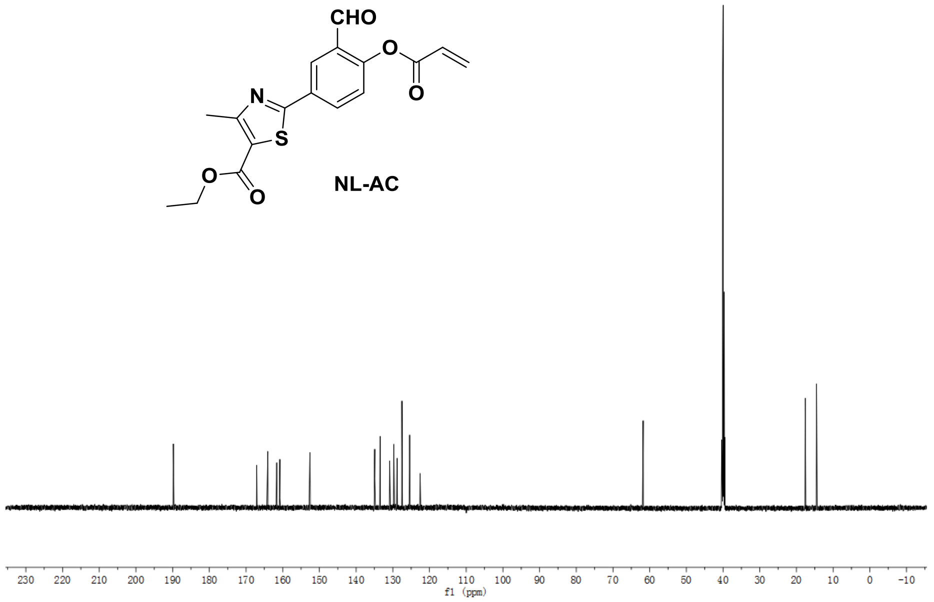

Figure 1. NMR Spectra for **NL-AC**.

UPLC-MS spectrum of NL-AC + Cys

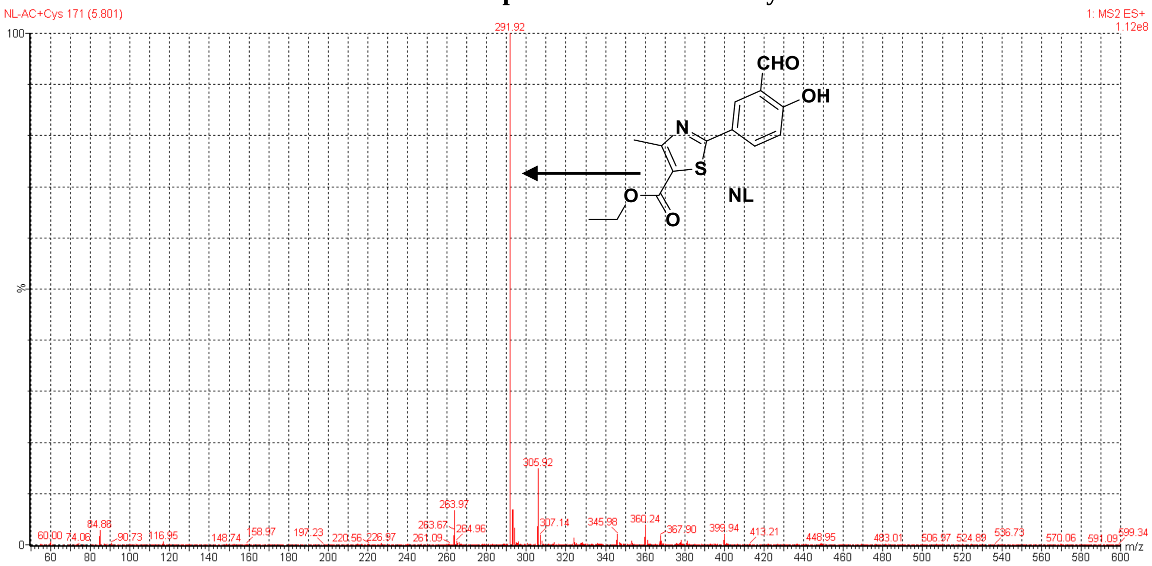

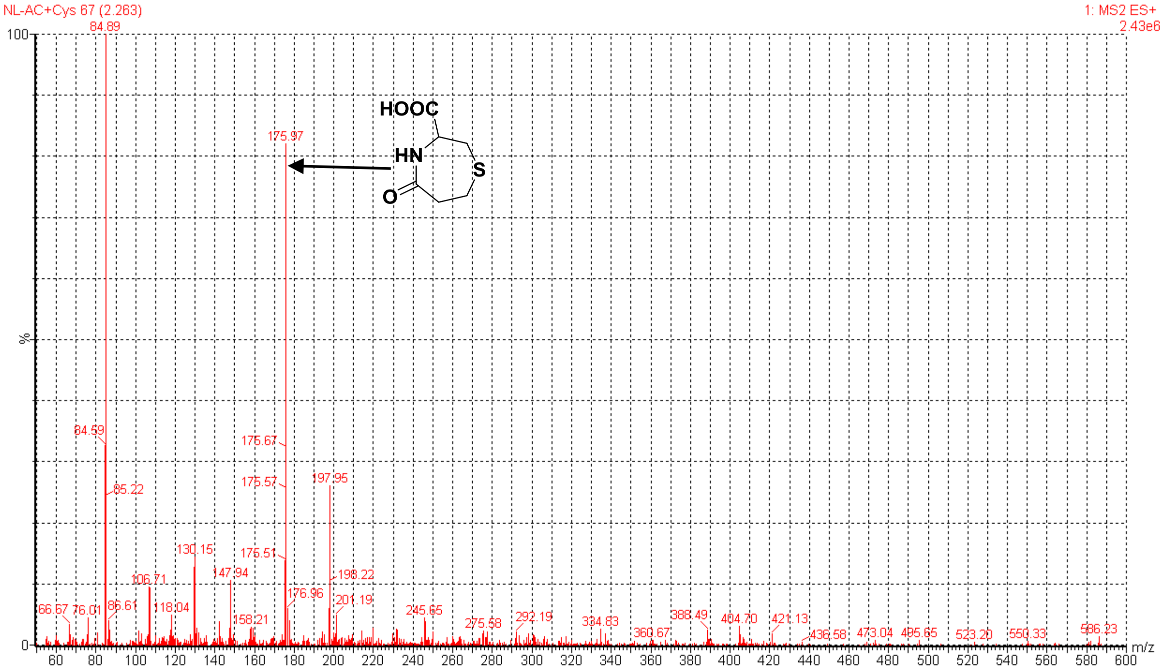

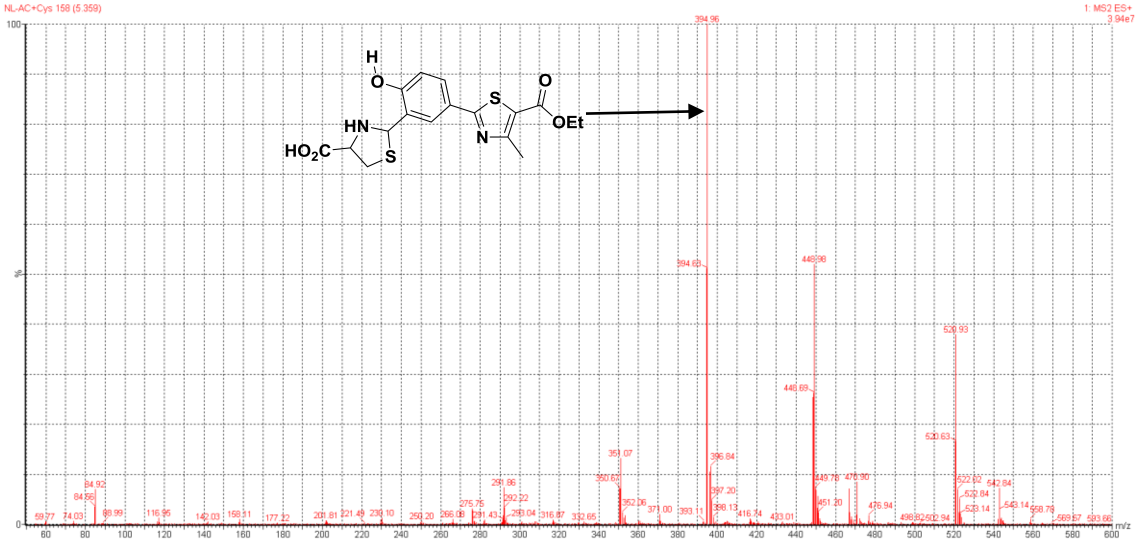

UPLC-MS spectrum of the products of the reaction between NL-AC and Hcy

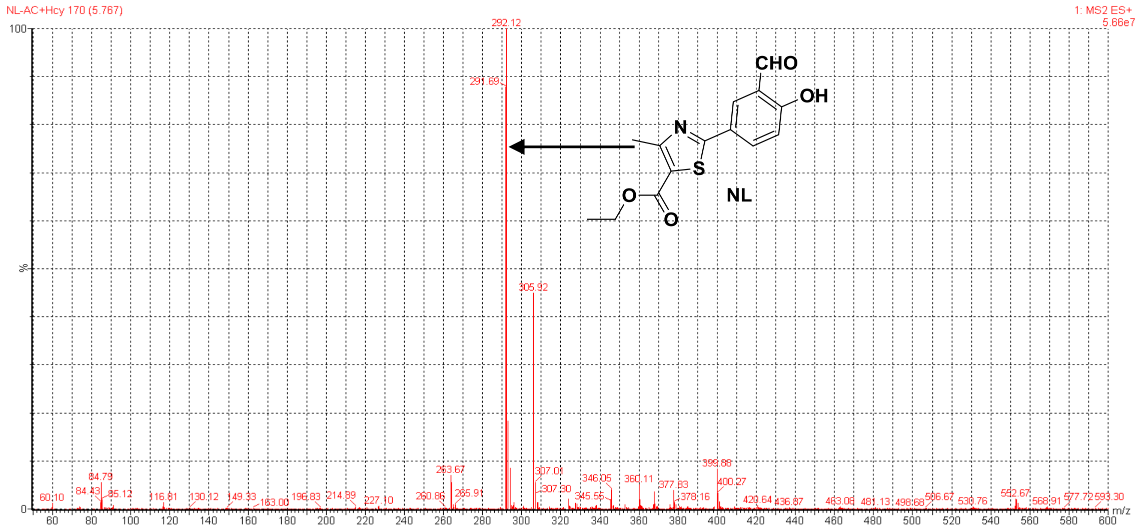
NL-AC+Hcy 170 (5.767)
1: MS2 ES+
5.66e7
292.12
291.69
305.92
CHO
OH
N
S
O
O
NL
84.79
84.43
85.12
60.10
116.81
130.12
149.33
163.00
196.83
214.99
227.10
260.86
263.67
265.91
307.01
307.30
345.56
346.05
360.11
377.83
378.16
399.88
400.27
420.64
436.87
463.08
481.13
496.68
506.67
538.76
552.67
568.91
577.72
593.30
%
m/z

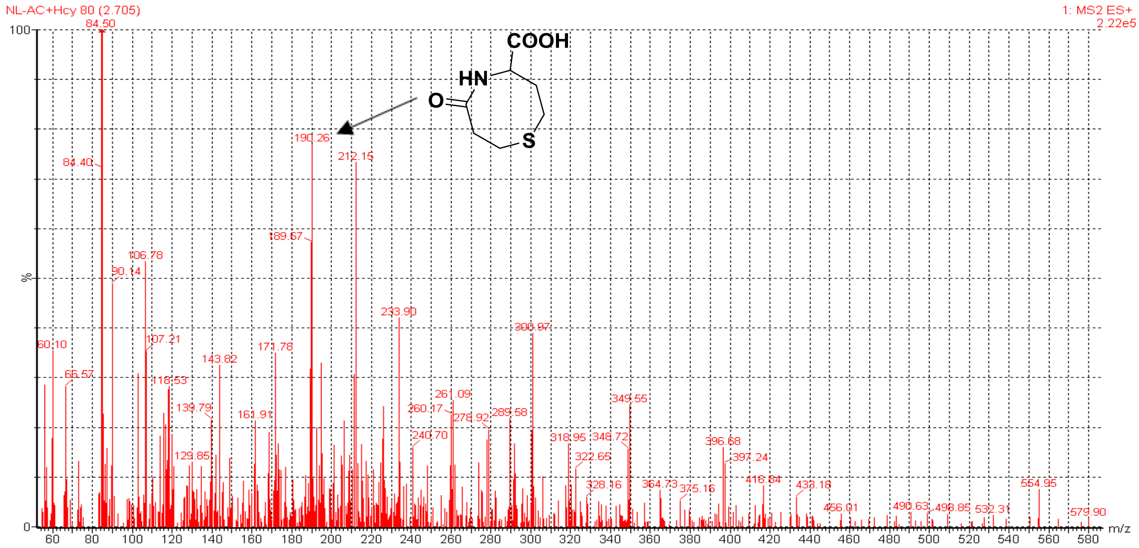
NL-AC+Hcy 80 (2.705)
1: MS2 ES+
2.22e5
84.50
84.40
COOH
HN
O
S
190.26
212.15
189.67
106.78
90.14
107.21
60.10
66.57
118.53
143.82
171.78
233.90
300.97
139.79
161.91
129.85
261.09
260.17
278.92
289.58
240.70
349.55
318.95
346.72
322.65
328.16
364.73
375.16
396.68
397.24
416.84
433.18
456.01
490.63
498.85
532.31
554.95
579.90
%
m/z

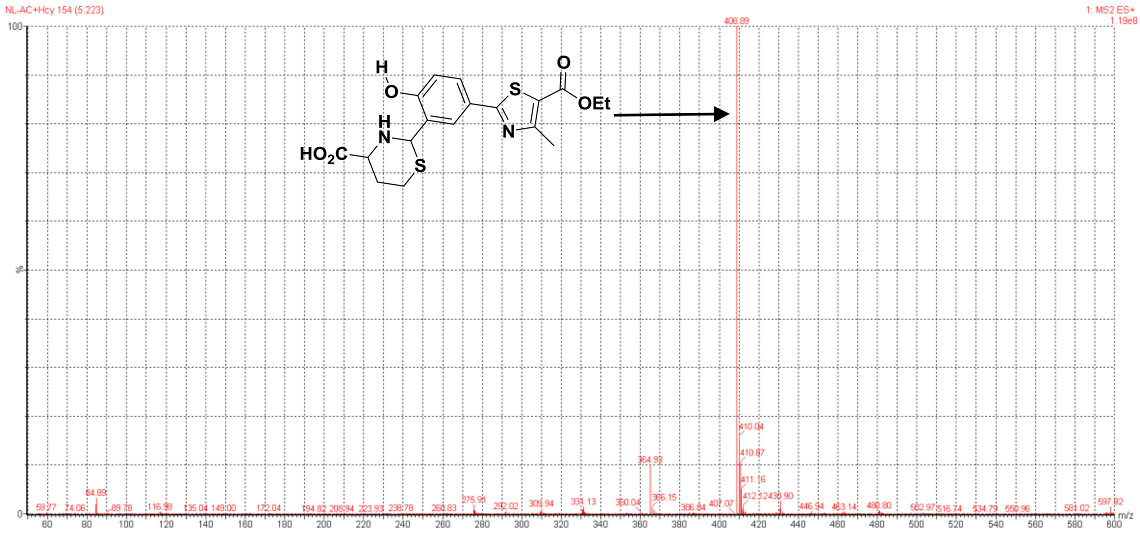
NL-AC+Hcy 154 (5.223)
1: MS2 ES+
1.19e8
408.89
H
O
S
O
OEt
N
H
N
HO2C
S
410.04
410.67
411.16
364.93
366.15
84.89
59.77
74.06
89.28
116.98
135.04
149.00
172.04
194.82
208.94
223.93
238.78
260.83
275.91
292.02
309.94
331.13
350.04
386.84
402.07
412.32
430.90
446.94
463.14
480.80
502.97
516.74
534.79
550.96
581.02
597.92
%
m/z

UPLC-MS spectrum of NL-AC + GSH

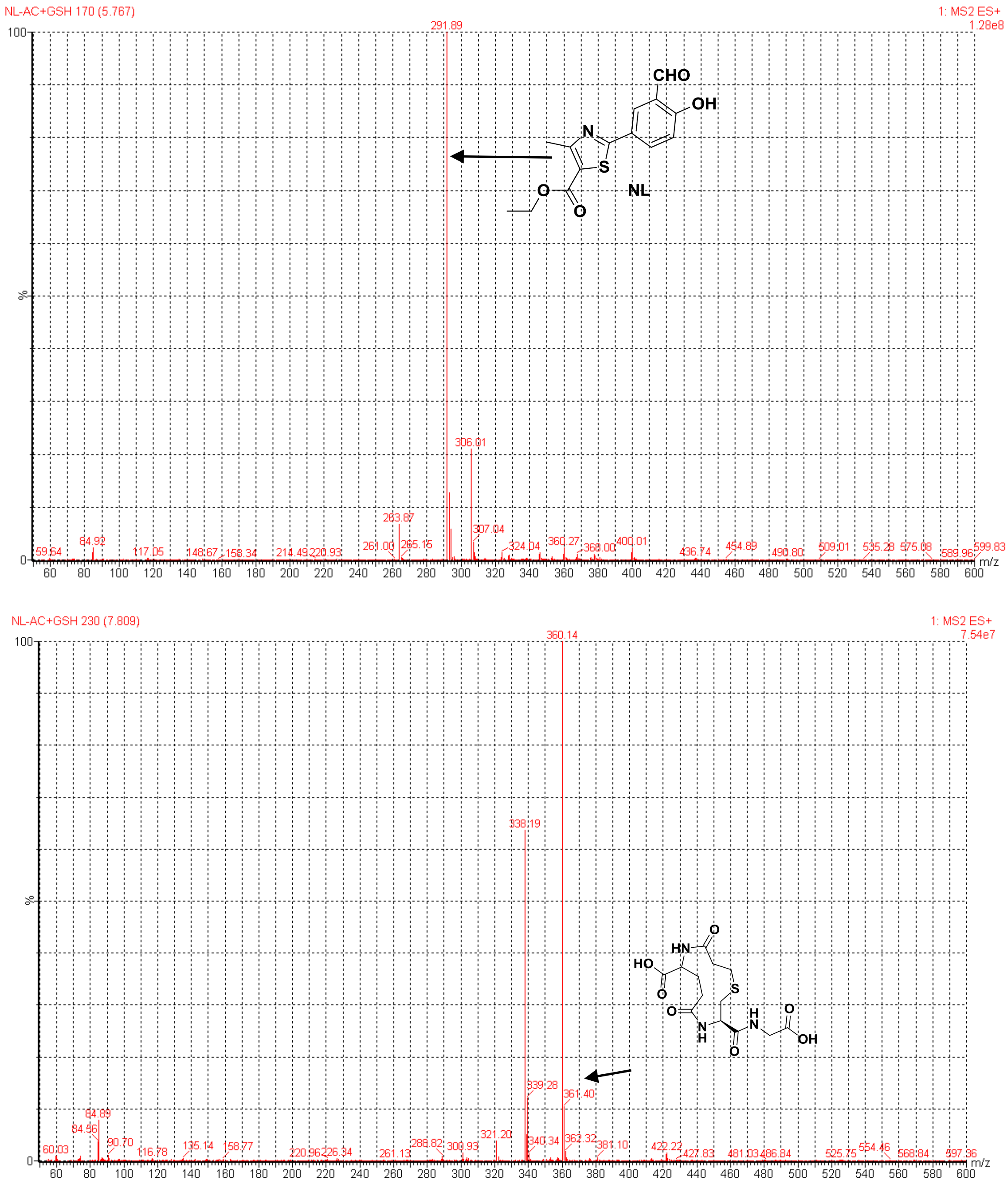

Figure 2. UPLC-MS spectra of **NL-AC**, **NL-AC** + Cys, **NL-AC** + Hcy and **NL-AC** + GSH.

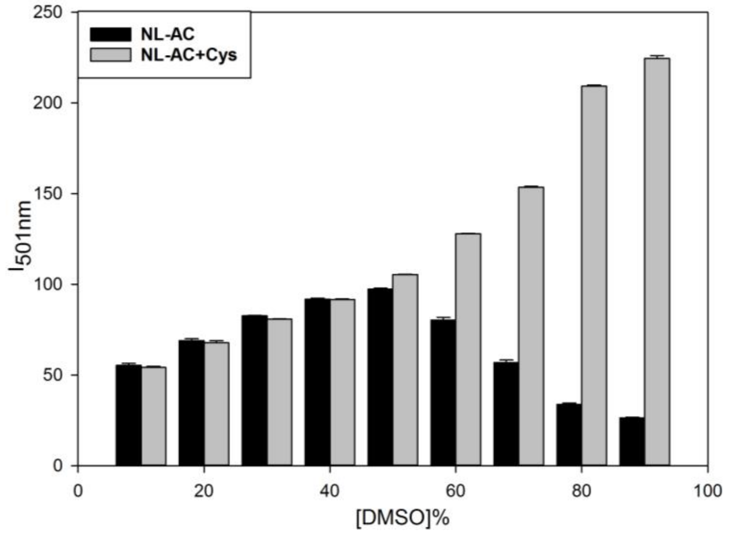

Figure 3. Fluorescence response of **NL-AC** (1×10^{-5} M) without (black) and with (gray) Cys (2×10^{-4} M) in HEPES buffer solution with different ratios of DMSO and HEPES. (pH 7.4, λ_{ex} = 336 nm, slit: 5.0 nm/5.0 nm).

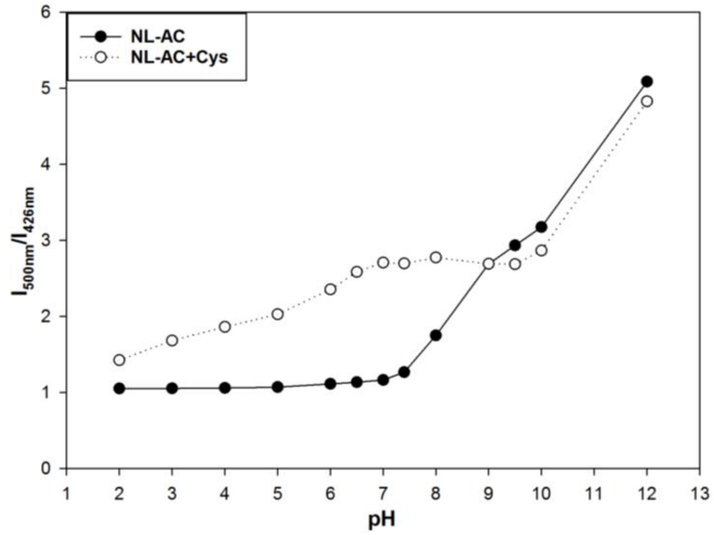

Figure 4. Ratiometric responses of **NL-AC** (1×10^{-5} M) without or with Cys (2×10^{-4} M) in HEPES buffer solution (DMSO/HEPES = 8:2, λ_{ex} = 336 nm, slit: 5.0 nm/5.0 nm) at different pH

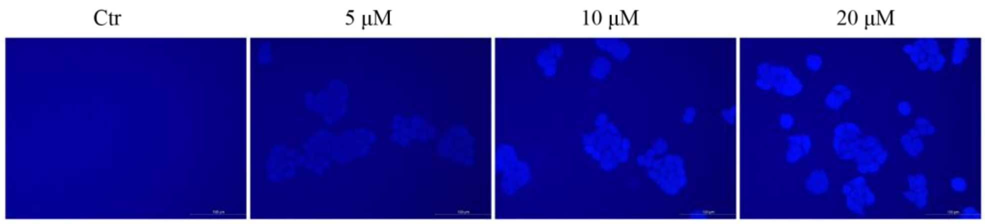

Figure 5. Fluorescence images of the probe **NL-AC** in HeLa cells with different concentrations.

Table 1. The summary of fluorescence probes for the detection of biothiols

**Probes**	**Fluorophore**	**$\lambda_{ex}/\lambda_{em}$ (nm)**	**Ratiometric or not**	**Response time**	**Detection limit**	**Target**	**Samples**	**Ref.**
NL-AC	HBT	336/426 336/501	yes	20 min	0.1 μM	Cys, Hcy and GSH	HeLa cells	This work
NL	HBT	327/426 327/501	yes	150 min	0.9 μM	Cys and Hcy	/	Na et al., 2016 [1]
C-3	Cyanine	580/640,	yes	5 min	0.2 μM	Cys, Hcy and GSH	HeLa cells	Bab ür et al., 2016 [2]
NSTTA-Eu3^{+}/Tb3^{+}	NSTTA	328/540， 328/610	yes	800 s		Cys, Hcy and GSH	HeLa cells	Dai et al., 2013 [3]
Probe 1	N-Butyl-4-amino-1,8-naphthalimide	420/482， 420/540	yes	120 min	3.7 μM	Cys, Hcy and GSH	HeLa cells	Gao et al., 2016 [4]
Probe 1	Aminocoumarine	345/400 345/440	yes	600 min	6.0 μM	Cys, Hcy and GSH	/	Lim et al., 2013 [5]
Probe 1	Benzoxazine-hemicyanine	496/570 496/679	yes	/	/	Cys, Hcy and GSH	KB cells	Liu et al., 2013 [6]
QMA	Quinoline α,β-unsaturated diacid	360/525	no	40 min	/	Cys, Hcy and GSH	MDA-MB 231 cells	Song et al., 2013 [7]

"/" means not mentioned

References

1. Na, R. *et al.* A Simple and Effective Ratiometric Fluorescent Probe for the Selective Detection of Cysteine and Homocysteine in Aqueous Media. *Molecules* **21**, 1023-1031, doi:10.3390/molecules21081023 (2016).
2. Babur, B. *et al.* A novel fluorescence turn-on coumarin-pyrazolone based monomethine probe for biothiol detection. *Tetrahedron* **72**, 4498-4502, doi: 10.1016/j.tet.2016.06.008 (2016).
3. Dai, Z. *et al.* A Lanthanide Complex-Based Ratiometric Luminescence Probe for Time-Gated Luminescence Detection of Intracellular Thiols. *Analytical Chemistry (Washington, DC, United States)* **85**, 11658-11664, doi: 10.1021/ac403370g (2013).
4. Gao, B. *et al.* A highly selective ratiometric fluorescent probe for biothiol and imaging in live cells. *RSC Adv.* **6**, 43028-43033, doi: 10.1039/c6ra04564b (2016).
5. Lim, S.-Y. *et al.*. 7-Aminocoumarinyldisulfide as a ratiometric fluorescent probe for biothiols in water. *Sensors and Actuators, B: Chemical* **185**, 720-724, doi: 10.1016/j.snb.2013.05.053 (2013).
6. Liu, X.-D. *et al.* A benzoxazine-hemicyanine based probe for the colorimetric and ratiometric detection of biothiols. *Sensors and Actuators, B: Chemical* **178**, 525-531, doi: 10.1016/j.snb.2012.12.108 (2013).
7. Song, Q.-H. *et al.* A novel fluorescent probe for selective detection of thiols in acidic solutions and labeling of acidic organelles in live cells. *Journal of Materials Chemistry B* **1**, 438-442, doi: 10.1039/C2TB00402J (2013).
